# Toward Clarity in Single Extracellular Vesicle Research: Defining the Field and Correcting Missteps

**DOI:** 10.1021/acsnano.5c00705

**Published:** 2025-04-24

**Authors:** Yun Su, Wanzhuo He, Lei Zheng, Xianqun Fan, Tony Y. Hu

**Affiliations:** † Department of Ophthalmology, Shanghai Key Laboratory of Orbital Diseases and Ocular Oncology, Shanghai Ninth People’s Hospital, Shanghai Jiao Tong University School of Medicine, Shanghai 200011, P. R. China; ‡ Department of Laboratory Medicine, Guangdong Provincial Key Laboratory of Precision Medical Diagnostics, Guangdong Engineering and Technology Research Center for Rapid Diagnostic Biosensors, Guangdong Provincial Key Laboratory of Single Cell Technology and Application, Nanfang Hospital, Southern Medical University, Guangzhou 510515, P. R. China; § Department of Biochemistry and Molecular Biology, Center for Cellular and Molecular Diagnostics, Tulane University School of Medicine, New Orleans, Louisiana 70112, United States

**Keywords:** Extracellular vesicles (EVs), Single EV research, Heterogeneity, EV characterization, Biological function, Diagnostics, Therapeutics, Precision medicine

## Abstract

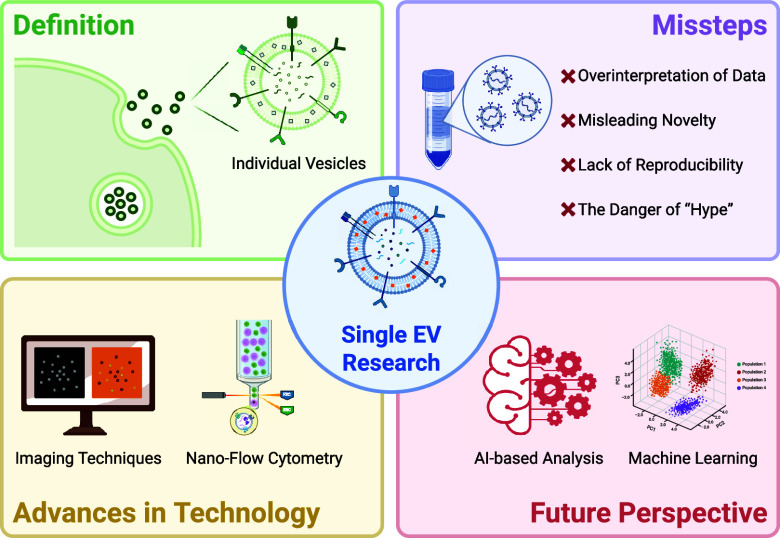

Single extracellular vesicle (EV) research holds the potential to revolutionize our understanding of cellular communication and enable breakthroughs in diagnostics and therapeutics. However, the lack of a clear, consensus-driven definition of single EV research has led to methodological inconsistencies, overgeneralized interpretations, and, in some cases, misleading claims. In this perspective, we propose a framework for defining single EV research, critique current challenges and misconceptions in this field, and discuss its implications for biomedical applications. We argue that precise experimental design, rigorous validation, and interdisciplinary collaboration approaches are needed to establish single EV research as a cornerstone of precision medicine.

## 1 Introduction

Extracellular vesicles (EVs) are increasingly recognized as powerful mediators of intercellular communication, that carrying diverse cargoes, including proteins, lipids, and nucleic acids. Their potential as biomarkers and therapeutic vectors has propelled the field forward. However, bulk EV analysis often masks the heterogeneity inherent in EV populations, leaving critical subpopulations—and their specific roles—undiscovered.

The emergence of single EV research promises to fill this gap since, by enabling the characterization of individual vesicles, it offers unprecedented insight into EV heterogeneity and biology. However, the field has struggled with foundational issues: What defines single EV research? How should we interpret single EV data in the context of bulk findings? What are the limitations of current approaches?

The lack of clarity in addressing these questions has led to significant challenges. Misleading studies, which stem from unclear definitions or overinterpretation of data, risk undermining the field’s credibility. This perspective aims to critically evaluate these issues, provide a clear definition of single EV research, and propose a roadmap to address current challenges.

## 2 Defining Single EV Research: A Necessity for Progress

### 2.1 The Current State of Definitions

The term “single EV research” is often used loosely, encompassing studies that range from population-level inferences based on limited single-particle data, to detailed, multimodal analysis of individual vesicles. This inconsistency has caused confusion, with some studies claiming “single EV” insights while relying on low-resolution methods that are unable to isolate true single-vesicle properties. This lack of clarity and standardization undermines the comparability and reproducibility of research findings, making it difficult for the scientific community to build a cohesive understanding of EV biology and function.

### 2.2 Toward a Precise Definition

**Figure 1:**
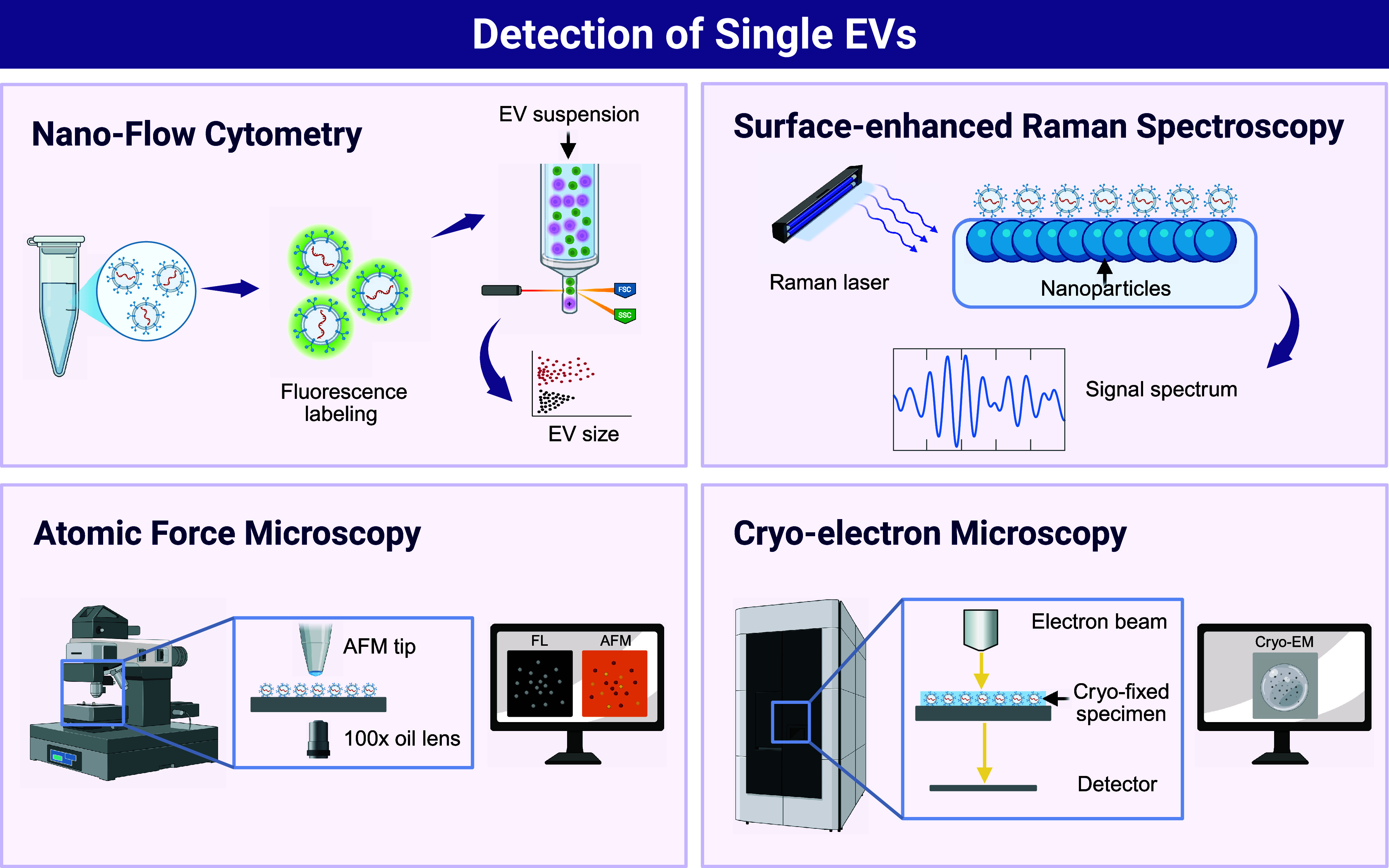
Representative techniques for detecting single EVs. Advanced methodologies employed for the detection and characterization of single EVs are illustrated. Nanoflow cytometry enables high-throughput analysis and quantification of surface markers on EV populations with minimal sample consumption. Surface-enhanced Raman spectroscopy (SERS) provides detailed chemical composition information by amplifying vibrational signals from EV surface molecules. Atomic force microscopy (AFM) offers high-resolution imaging and measurement of mechanical properties, revealing the nanoscale morphology and stiffness of single EVs. Cryo-electron microscopy (Cryo-EM) allows visualization of EVs in their native, frozen-hydrated state, providing structural insights into their complex architecture. These techniques collectively offer a comprehensive way for understanding the biology of single EVs. Created in BioRender; Su, Y. (2025), https://BioRender.com/r1uhiq4.

We propose that single EV research be defined by the following criteria, which collectively ensure the precision, depth, and relevance of the studies conducted (Figure 1):

#### 2.2.1 Physical Isolation or Detection of Individual Vesicles

True single EV studies should either directly analyze individual EVs or employ high-resolution, quantitative approaches that are capable of reliably extrapolating the properties of individual EVs. This emphasizes the need for precise, individual EV analyses, rather than relying on aggregate data from bulk EV populations, which can obscure the unique characteristics of individual vesicles.

An increasing number of techniques have been developed to permit the comprehensive and accurate analysis of EVs, each with its own unique advantages and limitations.^1^ For instance, high-sensitivity nanoflow cytometry (nFCM)^2−4^ represents significant advances that enable high-throughput analysis of single EVs based on their specific fluorescence and scattering properties. These approaches allow researchers to accurately characterize EV subpopulations and detect rare events within complex mixtures, thereby providing valuable insight into the functional diversity of EVs.

On the other hand, many microfluidic devices can offer robust platforms for the isolation, capture, and molecular profiling of single EVs.^5^ These devices are designed to handle minute volumes of samples and can efficiently separate EVs from other cellular components to facilitate analysis of their heterogeneity and biological functions. By enabling the isolation and analysis of single EVs, microfluidic devices help to bridge the gap between bulk analysis and single-particle studies.

Advanced imaging techniques, such as Raman tweezers spectroscopy (RTM)^6,7^ and atomic force microscopy (AFM),^8,9^ also provide unprecedented nanoscale resolution that permits the direct visualization and characterization of single EVs. These techniques allow researchers to observe the morphology, size, and surface properties of single EVs in detail to enhance our understanding of their structure and function. By combining these imaging tools with other analytical methods, researchers can gain a more comprehensive view of EVs and their role in biological processes.

Collectively, these techniques represent a powerful suite of tools for the isolation, analysis, and interrogation of single EVs with minimal ambiguity, and thus provide a solid foundation for single EV analysis and have the potential to revolutionize our understanding of EV biology and specific roles of EVs in disease progression and treatment.

#### 2.2.2 Rigorous Multimodal Characterization

Single EV studies should integrate diverse analytical methods to capture the comprehensive physical and molecular uniqueness of distinct vesicles. To achieve this, it is crucial to employ high-resolution imaging techniques. This could include cryo-electron microscopy (cryo-EM), which can permit nanometer-resolution of EV morphology and membrane structures.^10^ Atomic Force Microscopy (AFM) can also enable the measurement of mechanical properties and surface topology of these vesicles under physiological conditions.^8^ Such imaging methods are essential for visualizing the intricate details of vesicle morphology and ultrastructure that may distinguish specific vesicle populations.

Molecular profiling techniques also provide indispensable means to assess EV cargoes and surface markers. These techniques encompass a wide range of methodologies, including Microfluidic-based Mass Spectrometry, Bead-based Immunocapture Assays, and Surface-enhanced Raman Spectroscopy (SERS), which can provide insight into the protein and lipid composition of EVs.^11−13^ Proximity Barcoding Assays (PBAs) can enhance such analyses by allowing more detailed protein profiling.^14^ Single-molecule RNA sequencing, digital PCR, and the use of microfluidic devices with next-generation sequencing approaches can serve as powerful means to characterize EV RNA cargoes,^15−17^ since these molecular profiling methods can provide a detailed assessment of their molecular contents. In addition to imaging and molecular profiling, functional assays could also be employed to evaluate the biological activities of single EVs and provide valuable information on the functional roles of EVs in various biological processes.

Integration of these complementary approaches–high-resolution imaging, molecular profiling, and functional analyses–would provide comprehensive EV characterizations necessary to generate a comprehensive picture of each vesicle’s distinct properties and capture its unique physical characteristics and the complexity of its molecular cargo. By employing this multifaceted approach, researchers could gain deeper insight into the roles and functions of EVs in processes that mediate homeostatic and disease processes.

#### 2.2.3 Validation of Biological Relevance

Observations made in single EV studies must be clearly linked to biologically or clinically meaningful questions to ensure that research findings are not merely descriptive but are instead interpretable in the context of broader biological or clinical phenomena. This should discourage overinterpretation of noise or artifacts in data sets and promote the translation of single EV insights into meaningful biological or therapeutic discoveries.

To achieve this, it is imperative that experimental designs be meticulously crafted to avoid potential misinterpretation of noise or artifacts. This requires the use of suitable statistical analyses and that validation studies be conducted in relevant biological models. For example, findings obtained from single EV profiling studies should not only be descriptive but should also be substantiated through functional studies that unequivocally demonstrate their impact of distinct EVs on specific target cells or tissues, as such studies are crucial to establish a clear link between these EVs and their biologically significant roles.

Correlations between EV characteristics and disease states or therapeutic responses should be firmly established using meticulously controlled clinical samples. However, it is important to recognize that EVs derived from most human biofluids are complex, containing information from a variety of cells and cell compartments. Accurately identifying changes in EV subpopulations is often indicative of changes in key biological processes. However, identifying these changes requires the use stringent methods and rigorous validation processes to ensure that any observed correlations are indicative of causal rather than coincidental relationships.

In summary, only by adhering to such a systematic and rigorous validation process can studies unequivocally confirm the biological significance of single EV measurements. This approach both enhances the credibility of research findings and facilitates the translation of these insights into practical applications that have the potential to improve human health and well-being.

This definition explicitly excludes studies that rely on ensemble averages of bulk EV populations or employ techniques that lack the sensitivity and specificity to distinguish individual vesicles from contaminants or cell debris. By adhering to these criteria, the field of single EV research can achieve a higher level of precision and rigor, leading to more meaningful and impactful discoveries.

## 3 Missteps in Single EV Research: Lessons Learned

### 3.1 Overinterpretation of Data

One common pitfall in the field of EV research has been the overinterpretation of data obtained from low-resolution techniques. This tendency can lead to misleading conclusions and hinder advances in our understanding of EV biology, as these methods may miss critical details about the structure and composition of EVs. Such methods also often lack the sensitivity and specificity required to accurately distinguish distinct types of EVs or to detect subtle changes in their molecular contents. Researchers using these approaches may therefore inadvertently overstate their conclusions about EV heterogeneity, size distribution, or the presence of specific markers, leading to a distorted view of EV biology and potentially misleading future research efforts.

#### 3.1.1 Nanoparticle Tracking Analysis (NTA)

NTA, which simultaneously measures both the size distribution and concentration of EVs in suspension, is a widely adopted technique for EV characterization.^18^ By leveraging light scattering and Brownian motion, NTA directs a laser beam through a glass prism onto EVs and captures the scattered light with an optical microscope to track the particles’ motion. This method provides detailed information across a wide range of EV sizes but requires precise calibration and parameter tuning for accurate data collection.^19,20^

While NTA is a highly valuable means of obtaining an overview of the bulk size distribution of EVs in a sample, but usually lacks sufficient resolution to reliably distinguish EVs from other similar-sized particles, such as lipoproteins or protein aggregates, when used as the sole support for claims about the properties of individual vesicles. This limitation arises because NTA primarily functions at the population level, averaging the characteristics of numerous EVs, which can lead to potential data misinterpretation if particulate contaminant skews the results and obscure the true nature of the EVs under study. Subtle differences in EV size, shape, surface markers, and other vesicle properties are also unlikely to be discernible using NTA alone, so that complementary analytical methods are often necessary to obtain a more comprehensive and detailed understanding of EV heterogeneity.

#### 3.1.2 Fluorescence-Based Approaches

Fluorescence-based approaches are the most prevalent means used to characterize single EVs due to their high detection sensitivity and the large number of fluorescent labels available for multiplex analyses.^21−23^ In these methods, EVs are typically immobilized on a coverslip or within a microfluidic chip by antibody/aptamer-based capture or nonspecific adsorption processes, and then labeled with fluorescent probes to permit their visualization and detailed examination of their components.^24^

To improve the signal-to-noise ratio (SNR) of these measurements, many studies have employed total internal reflection fluorescence (TIRF) imaging approaches,^25−27^ or highly fluorescent nanoparticle labels.^28−30^ Some studies also mention the utilization of diverse signal amplification mechanisms, such as rolling cycle amplification,^31^ to enhance imaging contrast. However, it is important to acknowledge that a SNR increase may sometimes compromise the specificity of detection. Given that the efficiency of the amplification reaction can vary between single EVs, some original information on EVs may be lost during the amplification process. Single-vesicle fluorescence data is also often reported without adequate consideration for false positives that may arise from dye aggregation or background noise. Further, the use of fluorescent labels may also alter the physical and biological properties of the EVs, thereby introducing another layer of complexity and potential bias into the analysis. Such oversights may lead to potential misinterpretation of results and the risk of overstating conclusions regarding the heterogeneity and molecular content of the analyzed EVs. By failing to account for these potential artifacts, researchers may draw premature or inaccurate inferences about the properties and functions of specific EVs and undermine the reliability and validity of their findings.

### 3.2 Misleading Claims of Novelty

Another significant issue arises when studies claim groundbreaking EV insights without employing adequate controls. For instance, the crucial task of distinguishing true EV cargo from coisolated contaminants, such as protein aggregates, lipoproteins, and other non-EV components, is often neglected in the rush to publish novel findings. The purity and yield of EVs obtained by different techniques may markedly vary among different sample types, as these EV isolate characteristics are largely dependent on their principles of the isolation method and the intrinsic composition of the starting sample. EV separation methods may thus exhibit differential performance when applied to different sample types.

For example, ultracentrifugation can eliminate most contaminants from many sample types, but it has a tendency to cosperate Tamm-Horsfall protein (THP) and EVs from other contaminants. The potential effects of THP-EV complex on downstream analyses remain unknown. Similarly, polymer-based precipitation methods are commonly used for EV isolation, but residual polymers present in EV isolates could potentially affect the observation of biological effects. Notably, some studies have reported that contaminants coisolated with EVs are the primary source of effects observed with EV isolates. Precipitation and filtration-based EV isolation methods, such as ExoDisc, may exhibit superior performance when employed with samples that contain fewer particulate contaminants.^32,33^ Researchers should therefore select their EV separation method after considering the sample type, the intended downstream analyses, and specific conditions related to their experimental design. Failing to account for potential confounding factors that may arise from these choices may lead to contaminated samples and misinterpretation of the results produced from them. This can lead to the publication of spurious conclusions about the molecular composition or the functional roles of the isolated EVs, misleading the scientific community and hindering progress in understanding the complex biology of these important cellular mediators.

### 3.3 Lack of Reproducibility

EVs secretion is influenced by a variety of biological and physical factors, and variations in EV isolation and analytical techniques has contributed to irreproducible EV research findings. Some of this variability arises from the wide range methods and protocols employed for EV isolation, which can lead to substantial differences in the composition and purity of the isolated EVs. For example, differential centrifugation, which is widely used for EV isolation, separates particles based on their size, density, and sedimentation coefficients under centrifugal force.^34^ However, despite its popularity, results obtained by this method can vary with differences in the starting biological sample, centrifugation parameters, contaminants, and other technical differences, and this can pose significant challenges for achieving consistent and reproducible results.^35−37^

Many studies fail to report critical information, including details of the specific protocols used for culture or specimen isolation, EV isolation procedures, instrument calibration procedures, and statistical validation methods, which can all be essential to ensure the reproducibility of their findings. Lack of transparency and standardization in reporting these details hinders the ability of other researchers to replicate results and build upon existing knowledge. Addressing this issue will require a concerted effort to develop and adopt standardized EV isolation, characterization, and analysis protocols and reporting guidelines to facilitate consistent and reliable reporting of EV research results.

### 3.4 The Danger of “Hype”

The rapid increase in interest regarding EV research and its applications has occasionally fostered a “hype” culture, where potential applications of single EV studies are exaggerated without sufficient initial experimental evidence or subsequent validation studies. Surges in interest may be fueled by the potential for groundbreaking discoveries and novel therapeutic applications and may be coupled with new advances in EV isolation, characterization, and high-throughput sequencing methods, or advanced analytical techniques. Market demand and commercialization efforts have accelerated the pace of EV research by attracting a substantial amount of investment. Media attention and increasing public awareness have also contributed to a broader understanding and appreciation of the potential of EV research, fostering a supportive environment for ongoing efforts. While this excitement presents opportunities for innovation and commercialization, it also requires a balanced approach to ensure scientific rigor and responsible development.

Despite there now being little EV research that can be readily translated into practical clinical applications, preliminary findings are sometimes presented as definitive breakthroughs in the current environment, leading to overinflated expectations and misplaced enthusiasm. While enthusiasm and a sense of urgency in advancing the state of art for the field are undoubtedly important drivers of progress, they must be carefully balanced with scientific rigor and a commitment to thorough experimentation. It is thus important that the EV research field preserve its credibility and build a strong foundation for future discoveries and innovations by developing and maintaining a rigorous approach to EV research and ensuring that reported claims are supported by robust data.

## 4 Organizing Single EV Research: A Roadmap

### 4.1 Technological Innovations

**Figure 2:**
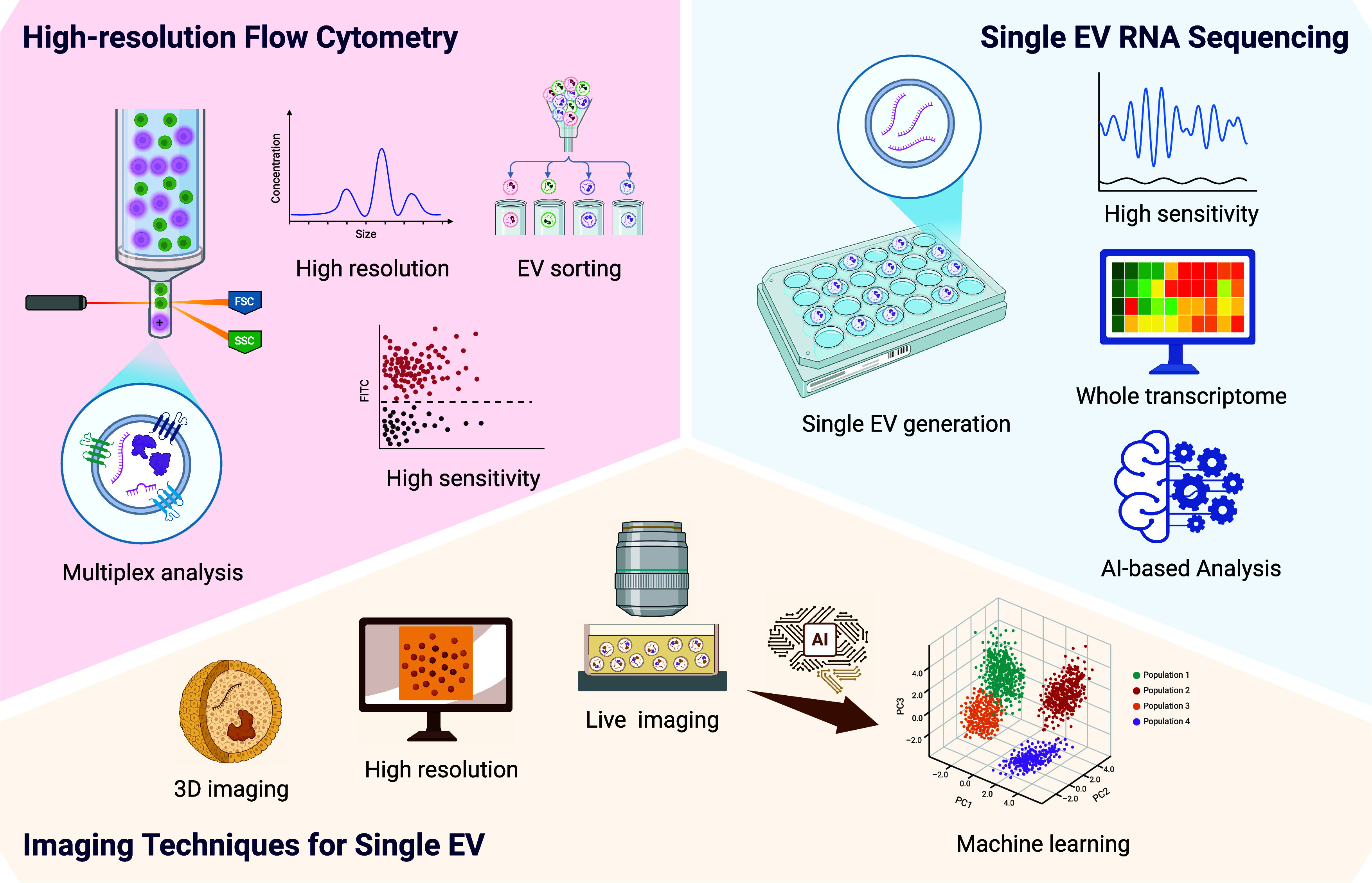
Technological innovations in single EV studies. This figure showcases the advanced tools essential for the accurate and reliable analysis of single EVs in the future. These technological innovations offer high sensitivity and specificity, enabling detailed investigation into the complex biology of EVs. By employing cutting-edge methodologies, researchers can gain deeper insights into the roles of EVs in physiological and pathological processes. These tools are pivotal for advancing our knowledge of EV function and their potential applications in diagnostics and therapeutics. Created in BioRender; Su, Y. (2025), https://BioRender.com/vg20k0z.

Single EV studies require advanced tools capable of high sensitivity and specificity analyses to ensure accurate and reliable results, and which can provide deeper understanding of the complex biology of EVs and their roles in physiological and pathological processes. Some of the promising technologies that meet these criteria include (Figure 2):

#### 4.1.1 High-Resolution Flow Cytometry

is indispensable for phenotyping rare EV subpopulations, since this method leverages optic and fluid dynamics principles to analyze EVs in a high-throughput manner. High-resolution flow cytometry can differentiate EVs with subtle differences in their surface markers, including proteins, lipids, and carbohydrates,^38−40^ by laser-illuminating single EVs and detecting signal emitted by fluorescent reporters bound to these EVs. The discriminatory capacity of this approach is crucial for identifying and characterizing rare EV subpopulations that may have distinct functional properties and biological roles. By providing valuable insights into the functional heterogeneity of EVs, high-resolution flow cytometry enables researchers to gain a deeper understanding of the complex biology of EV populations and identify specific EV subtypes that may have potential therapeutic applications.^41^

In the future, multiplex analysis will play a pivotal role in enhancing the ability to simultaneously detect multiple surface markers on EVs, providing a more comprehensive view of their phenotypic profiles. This will be achieved through advancements in spectral imaging and multilaser systems, allowing for the integration of a broader range of fluorescent reporters. Moreover, the pursuit of higher resolution will continue to drive technological innovations. By refining the optics and detection systems, researchers will be able to achieve even finer details in the characterization of EV surface markers, further distinguishing subpopulations with minute differences. High sensitivity will also be a critical area of focus. As the sensitivity of flow cytometry instruments improves, it will become possible to detect and analyze EVs at lower concentrations, enabling studies of EVs in biological fluids where they are naturally present in minute quantities. Lastly, EV sorting will emerge as a powerful tool for isolating specific EV subpopulations based on their phenotypic profiles. This will involve the development of sophisticated sorting algorithms and high-speed sorting mechanisms, enabling researchers to purify EVs with specific functional properties for further downstream analysis or therapeutic applications.

#### 4.1.2 Atomic Force Microscopy (AFM) and Cryo-Electron Microscopy

are essential for EV structural analysis studies as they offer unique insights into their physical characteristics and composition. AFM operates by measuring the forces between a nanoscale probe and the sample surface, enabling researchers to achieve nanometer-scale resolution of surface features.^42,43^ The high resolution of AFM allows the visualization of the intricate surface morphology and mechanical properties of single EVs, and can characterize their shape, size, and surface roughness. The ability of AFM to map EV surfaces with nanometer precision can reveal valuable information about the arrangement of EV surface factors and their interactions that can be important for understanding the behavior and function of EVs, including specific EV subtypes.^44^

Cryo-electron microscopy preserves the native state of EVs by rapidly freezing them at cryogenic temperatures, typically below −150 °C, and imaging them at high resolution. The rapid freezing approach used by this technique minimizes the potential for artifacts to form during the freezing process to ensures that EVs are imaged in a state that closely mimics their physiologic condition.^45^ Cryo-electron microscopy images can achieve resolutions of < 4 Å, allowing researchers to visualize detailed EV structural features, including the arrangement of proteins, lipids, and other biomolecules within their membranes and interiors.^46^ This technique can provide an unprecedented degree of insight into the structural complexity and functional diversity of EVs by employing high resolution electron microscopy to visualize cryopreserved structures that reflect their native state on EVs.

Future developments in these imaging techniques will further enhance their capabilities. Both AFM and cryo-electron microscopy will continue to push the boundaries of resolution. AFM may incorporate advanced probe designs and more sensitive force measurement techniques to achieve atomic-scale resolution, enabling even more detailed studies of EV surface structures. Cryo-electron microscopy, with ongoing advancements in detector technology and image processing algorithms, will strive for resolutions approaching the molecular level, offering deeper insights into the fine structures of EV components. Besides, integration of artificial intelligence (AI) will transform these imaging techniques into high-throughput platforms. AI algorithms can automate image acquisition, processing, and analysis, significantly reducing the time required for EV structural studies. Machine learning models can be trained to identify and classify EVs based on their structural features, facilitating large-scale EV characterization studies. In addition, the development of three-dimensional (3D) imaging capabilities will be a major milestone. AFM can be extended to 3D imaging by scanning the sample surface in multiple planes, while cryo-electron tomography will enable the reconstruction of 3D structures of EVs from a series of 2D images. These 3D images will provide a comprehensive understanding of the spatial organization and interactions within EVs. Moreover, achieving real-time imaging of single EVs will ultimately be a revolutionary breakthrough. Although it remains a significant challenge at present, with continuous advancements in technology, such as the development of novel labeling techniques and the emergence of more sensitive detection systems, it will become possible to observe the dynamic behavior and structural changes of individual EVs in real-time. This will allow researchers to directly observe the functional activities of EVs in physiological environments, such as membrane fusion and cargo release, thereby revealing the precise mechanisms of EVs in intercellular communication.

#### 4.1.3 Single-EV RNA Sequencing

provides vesicle-level transcriptomic insights by profiling of the RNA cargoes of single EVs to offer unprecedented detail about the RNA content and potential functional role of individual EVs from specific EV subpopulations.^47^ Single-EV RNA sequencing thus captures the heterogeneity and complexity of RNA species present within individual EV, allowing this information to be correlated with specific EV characteristics, unlike traditional bulk sequencing methods that provide averaged data from a large population of EVs that may demonstrate significant heterogeneity.

Future directions in this field include advancements in single EV generation techniques, enhancing the sensitivity of RNA sequencing to detect low-abundance transcripts, and expanding the analysis to the whole transcriptome. Efforts will be directed toward refining methods for isolating and analyzing single EVs with higher precision and throughput. This includes developing technologies for the generation of pure, intact single EVs from complex biological samples, ensuring accurate and reliable sequencing data. Advances in microfluidics and nanotechnology will play pivotal roles in these endeavors, enabling the isolation and manipulation of individual EVs with minimal disturbance. Second, enhancing the sensitivity of single-EV RNA sequencing will be crucial for detecting low-abundance RNA species. This may involve the development of more sensitive sequencing platforms and improved RNA amplification techniques. High sensitivity will facilitate a more comprehensive understanding of the RNA cargoes, including rare transcripts that may have significant functional roles in cell-to-cell communication and disease processes. In addition, expanding the scope of single-EV RNA sequencing to include whole transcriptome profiling will provide a more holistic view of the RNA content within EVs. This will encompass not only coding RNAs (such as mRNAs) but also noncoding RNAs (including miRNAs, lncRNAs, and other regulatory RNA species). Whole transcriptome analysis will enable a deeper exploration of the functional diversity and regulatory networks mediated by EV-associated RNAs.

By enabling the identification and quantification of RNA species within single EVs, these technologies offer crucial insights into the regulation of cell-to-cell communication, immune modulation, tissue repair, and other physiological processes. This has significant implications for improved understanding of roles EVs play in pathologic conditions, including the development and progression of cancer, inflammatory diseases, and neurodegenerative disorders. Better understanding of the RNA cargoes carried by specific EVs could facilitate the identification of new mechanisms of disease progression and new RNA biomarkers, therapeutic targets related to them. Single-molecule RNA sequencing can thus provide critical information to advance our understanding of EV biology and its critical roles in health and disease.

### 4.2 Standardization and Validation

Future advance in EV research will require the development and adoption of new research standards that improve the reliability and reproducibility of experimental results from EV studies. Some key areas that should be addressed in this effort include:

#### 4.2.1 Harmonized Protocols

To ensure the accuracy and reproducibility of research findings, the scientific community must adopt standardized methods for EV isolation, labeling, and analysis. This involves developing and widely disseminating protocols that have been rigorously tested and validated by multiple research groups. By harmonizing these protocols, researchers can minimize variability introduced by methodologic differences, which should produce more consistent results that are comparable results across different studies. Adopting standardized methods will also facilitate the translation of EV-based research from the bench to the bedside and facilitate the development of novel EV-based diagnostic and therapeutic applications.

#### 4.2.2 Cross-Platform Validation

Studies that directly compare different EV isolation, labeling, and analysis methods are required to establish the relative accuracy and robustness of results obtained with these techniques. Such studies involve applying multiple methods to the same sample sets and comparing the results to assess the consistency and accuracy of results obtained with each method. By conducting such cross-platform validation studies, researchers can identify potential biases and limitations of individual methods to guide refinement and optimization of existing protocols. Such studies are needed to ensure that the EV research community is utilizing the most accurate and reliable techniques available to enhance the overall quality and impact of EV-based research.

#### 4.2.3 Open Science Initiatives

Publicly accessible data sets annotated with EV isolation and analysis methods are essential to accelerate consensus-building and foster collaboration within the EV research community. By sharing raw data, processed data, and analytical tools, and detailed EV isolation methods researchers can validate each other’s findings, identify discrepancies, and collaboratively refine methodologies. Open science initiatives can also facilitate the discovery of new EV-based biomarkers, therapeutic targets, and mechanisms of action by enabling data integration and cross-study comparisons. By embracing open science principles, the EV research community could foster a culture of transparency, collaboration, and continuous improvement, which should drive progress in this rapidly evolving field.

### 4.3 Collaborative Networks

Interdisciplinary collaboration will be critical in overcoming technical and conceptual barriers in basic and applied EV research. Key initiatives that can foster such collaborations include:

#### 4.3.1 Global Consortia

Multi-institutional efforts aimed at benchmarking various tools and methodologies used in EV research, and validating findings across different studies, could play key roles in advancing the field. By working collaboratively, researchers from diverse institutions could pool their expertise and resources, enabling them to establish standards and protocols for EV isolation, purification, characterization, and analysis. Adherence to these guidelines would enhance the reliability and reproducibility of results from EV studies and foster greater confidence in the scientific community about the validity of findings from EV-based research studies. Such global consortia could also facilitate the sharing of data and samples, which would allow researchers to validate EV study findings across different patient populations, thereby strengthening the evidence base for EV-based diagnostics and therapies. Such concerted efforts would allow the global research community to more rapidly and consistently advance the rate of high-confidence discoveries in the field of EV research.

#### 4.3.2 Training Programs

Workshops and training programs should also be organized to develop the expertise necessary to foster advances in this rapidly evolving field. Such initiatives should be designed to provide EV researchers with in-depth knowledge of new and emerging technologies, best practices for EV isolation, purification, characterization, and analysis, as well as the latest advances in single EV diagnostics and functional studies. EV workshops should cover theoretical concepts, provide hands-on practical sessions, and discuss case studies to provide a holistic understanding of the state of EV research. EV training programs should focus on fostering a collaborative mindset among participants, encouraging them to share their expertise, insights, and challenges. By expanding the knowledge base and skill set of the research community, these programs should facilitate more effective and efficient collaboration, enabling researchers to tackle complex questions and develop innovative approaches to address them. Ultimately, these workshops and programs should contribute to efforts to accelerate the translation of EV-based discoveries into clinical applications to improve patient outcomes and advance global healthcare efforts.

#### 4.3.3 Regulatory Frameworks

Establishing clear and comprehensive guidelines is essential to facilitate the clinical translation of single EV diagnostics. Such frameworks should provide a roadmap for researchers, clinicians, and industry stakeholders, outlining the requirements to validate EV-based diagnostic tools and ensure their safety, efficacy, and reproducibility in a clinical setting. By creating a regulatory environment that not only supports innovation but also ensures rigorous scientific validation and adherence to high technical standards, these guidelines should pave the way for the widespread adoption and integration of single EV diagnostics into routine medical practice. Such regulatory frameworks should also promote international collaboration and technical alignments to facilitate the seamless exchange of knowledge, data, and technologies across borders. Establishing such regulatory frameworks should also ultimately accelerate the translation of promising EV-based research into clinically relevant diagnostics to improve patient care and advance the field of precision medicine.

### 4.4 Biomedical Potential of Single EV Research

#### 4.4.1 Contributions to Biology

Single EV studies offer unparalleled opportunities to explore various facets of EV processes, which can significantly advance understanding in several critical areas:

##### 4.4.1.1 Heterogeneity and Function

By precisely identifying specific EV subpopulations using advanced analytical techniques, researchers can uncover rare EVs that play unique and specific biological roles. These subpopulations may differ in size, shape, composition, and surface markers, and have distinct functions and effects within the biological milieu. The ability to dissect EV heterogeneity should allow a more nuanced understanding of the specific functions of distinct EV subsets and how they contribute to complex biological processes such as cell-to-cell communication, tissue repair, and immune modulation.^48^ By studying these EV subpopulations, researchers should gain additional insight into the regulatory mechanisms that govern EV biogenesis, trafficking, and clearance, which should ultimately lead to a more comprehensive understanding of EV biology and its effects on health and disease.

##### 4.4.1.2 Cargo Dynamics

Single EV characterization studies should also provide valuable insight into the selective packaging of biomolecules into EVs, or EV subpopulations. Further, an improved understanding of what factors are incorporated into EVs and specific EV subtypes, and how these correlates with other factors, should aid researchers in discovering underlying mechanisms that govern EV biogenesis and function. This degree of detail is crucial to aid in elucidating how specific EVs contribute to distinct biological processes, such as cell-to-cell communication and tissue homeostasis. More detailed information about the molecular cargoes of specific EVs should also aid scientists in identifying potential biomarkers or therapeutic targets for specific diseases and conditions. New findings from single EV characterization studies should thus not only advance our knowledge of EV biology but also pave the way for the development of innovative diagnostic tools and therapeutic strategies that harness the unique properties of EVs.

##### 4.4.1.3 Cellular Origins

High-resolution single EV analyses should also allow researchers to accurately trace target vesicles back to specific cell types, and this would be invaluable in efforts to advance understanding of the pathophysiology of specific diseases and chronic disease conditions. By pinpointing the cellular origins of EVs, researchers can also gain a better understanding of the potentially intricate EV-mediated interactions between cells and their microenvironments. This information could, in turn, foster the development of novel therapeutic strategies and diagnostic tools tailored to address specific mechanisms that regulate disease progression. High-resolution EV analysis studies are therefore likely to drive groundbreaking discoveries that could revolutionize the treatment and management of numerous medical conditions.

#### 4.4.2 Disease Diagnostics

Single EV analyses hold promise for revolutionizing disease diagnostics by offering a novel and highly sensitive means to detect and analyze disease-associated biomarkers that could facilitate personalized and precision medicine.

##### 4.4.2.1 Liquid Biopsies

Specific detection of rare disease-associated EV subpopulations in minimally or noninvasive biofluid specimens, including blood, urine, or cerebrospinal fluid, represents a promising approach to identify highly specific biomarkers for various diseases, like cancer, neurodegenerative diseases, and infections where it may not be feasible to directly obtain diagnostically useful biopsies of the diseased tissue.^49^ Disease-associated EV subpopulations that carry unique variants or combinations of proteins, lipids, and nucleic acids, can serve as powerful indicators of disease presence and progression. Over the past several decades, new analytical strategies (*e.g.,* microfluidic chips, nanowire arrays and electrochemical biosensors) have emerged as improved means for the rapid, accurate and high-throughput detection and analysis of target EVs, and have profoundly increased the potential feasibility of EV diagnostics that employ liquid biopsies.^50−52^

##### 4.4.2.2 Real-Time Monitoring

The dynamic nature of biofluid EV profiles renders them ideal candidates for real-time monitoring of disease progression or therapeutic efficacy. Changes in EV or EV subtype numbers and EV cargo composition can serve as early indicators of disease progression or the efficacy of a particular treatment. A variety of *in vivo* methods have therefore been employed to monitor such EV changes, including nuclear imaging, photoacoustic imaging, fluorescence imaging, and flow cytometry.^53−56^ However, while most of these detection methods can analyze *in vivo* EV distributions they cannot directly detect *in vivo* changes in EVs over extended periods, due to the transient nature of bioluminescence and radioactive markers. Furthermore, the nanoscale size of single EVs and their tendency to form agglomerates makes it challenging to achieve the same level of sensitivity and accuracy as can be achieved when detecting single cells. Novel real-time EV monitoring technologies that permit rapid and precise tracking and analysis of EVs are needed to provide new insights that can improve early disease detection and treatment, and rapidly and effectively monitor a disease’s response to treatment.

#### 4.4.3 Therapeutic Applications

Single EV research should also improve decision made in the development of new EV-based therapeutic applications. A deeper understanding of the diversity in EV size, composition, and function among different EV populations should allow research to design safer and more effective delivery systems for therapeutic agents. For example, EVs can be engineered to carry specific drugs or biomolecules directly to target cells based on their display of surface factors, bypassing the need for systemic administration of therapeutics, and minimizing their off-target effects. This targeted delivery approach has the potential to significantly enhance the efficacy and safety of a wide range of treatments, from cancer immunotherapy to gene therapy. Similarly, by harnessing the intrinsic properties of specific EVs, the field of regenerative medicine stands to make groundbreaking strides in developing innovative therapeutic strategies. However, both these applications require careful characterization of the EVs selected for these therapeutic approaches to avoid unintended consequences.

## 5 A Perspective on the Future

### 5.1 Moving Beyond “Hype”

The potential of single EV research is undeniably exciting as it promises to provide a deeper understanding of cellular communication and disease mechanisms and promote the development of new EV diagnostic and therapeutic applications. However, as with any rapidly developing field, it is crucial to temper research enthusiasm with a sense of realism to avoid the impression of overpromising from new discoveries. Rigorous, incremental progress is the cornerstone of scientific advances, and is necessary to ensure that the true potential of new EV research is realized without undermining the trust of the scientific community and the public.

### 5.2 Balancing Depth and Breadth

While pursuing new discoveries, it will be important to strike a balance between in-depth characterization of single EVs and broader analyses of their population-level dynamics. An exclusive focus on single EVs may provide granular insights, but it risks ignoring the broader context in which these vesicles operate. Conversely, overemphasizing population-level studies could obscure important effects or nuances contributed by single EV heterogeneity. Integrative approaches that link single EV findings to bulk studies are therefore imperative to maintain this balance. Such approaches should provide a comprehensive understanding of EV biology, capturing both the intricate details of individual vesicles and their collective behavior in biological systems.

### 5.3 Leveraging Emerging Technologies

The field of single EV research should harness the power of emerging technologies to evolve and progress. New advances in artificial intelligence (AI), microfluidics, and nanotechnology offer unprecedented opportunities to enhance single EV studies. For example:

#### 5.3.1 AI-Driven Data Analysis

AI approaches, particularly machine learning (ML) and large language models (LLMs), is poised to revolutionize single-EV research by addressing long-standing challenges in data interpretation, classification, and functional prediction. ML algorithms (*e.g.,* unsupervised clustering, graph-based neural networks) can analyze multimodal single-EV data sets (*e.g.,* Raman spectra, AFM mechanical properties, nanoflow cytometry profiles) to identify patterns and correlations in single EV data sets with remarkable speed and accuracy, including those that would be missed by conventional methods. Deep learning models (*e.g.,* CNNs for image-based EV detection, transformers for spectral data) trained on large EV libraries could enable real-time classification of EVs based on size, surface markers, or cargo (*e.g.,* miRNA, proteins), reducing reliance on antibody-based capture. ML can also correlate EV signatures with cellular origins or disease states (*e.g.,* tumor derived EVs) by integrating omics data, enhancing diagnostic and therapeutic discovery. In addition, LLMs (*e.g.,* GPT-4, BioBERT) can parse vast EV literature to extract mechanistic insights, predict EV-molecule interactions, or propose novel biomarkers by cross-referencing existing databases (*e.g.,* ExoCarta, Vesiclepedia). By training LLMs on EV protocols and experimental outcomes, researchers could optimize single-EV isolation workflows (*e.g.,* microfluidic chip designs) or troubleshoot technical artifacts. Taken together, harness AI for single-EV analysis should provide new insight into exosome biology and accelerate the development of novel diagnostic and therapeutic strategies.

#### 5.3.2 High-Throughput Platforms

New high-throughput platforms that employ miniaturized and automated systems have potential to revolutionize single EV analysis. By integrating modular microfluidics with automated liquid handling robotics, these systems could process thousands of EVs per hour while minimizing human intervention. Coupled with real-time AI-powered image analysis (*e.g.,* using convolutional neural networks for EV classification) and high-resolution mass spectrometry (such as Orbitrap or TIMS-TOF), such platforms could facilitate rapid, scalable, and multiparametric profiling of single EVs, from surface protein signatures to cargo composition. This leap in throughput of single EV studies could not only accelerate the translation of research findings into clinical applications but also uncover subtle EV-based signatures currently masked by bulk analysis. Critically, these platforms could facilitate the identification of novel biomarkers for specific diseases and chronic conditions by correlating EV heterogeneity with pathological states. Furthermore, by mapping EV-mediated intercellular communication networks, researchers could identify actionable therapeutic targets, such as EV-associated proteins or nucleic acids, that might be modulated by novel drugs, biologics (*e.g.,* engineered EVs), or RNA-based therapies. Ultimately, the convergence of these technologies could democratize single EV analysis, making it accessible for routine diagnostics and personalized medicine.

## 6 Conclusion

Single EV research represents a transformative frontier in EV research, but its utility will depend on clarity, rigor, and collaboration. Unlocking its full potential to advance basic and clinical research and to promote the development of new diagnostic and therapeutic applications will require that we address current EV research issues, adopt robust guidelines, and foster interdisciplinary partnerships. With a clear vision of what needs to be done and sustained effort, single EV research can move from a niche pursuit to a foundational pillar of precision medicine.
